# Generic HRTFs May be Good Enough in Virtual Reality. Improving Source Localization through Cross-Modal Plasticity

**DOI:** 10.3389/fnins.2018.00021

**Published:** 2018-02-02

**Authors:** Christopher C. Berger, Mar Gonzalez-Franco, Ana Tajadura-Jiménez, Dinei Florencio, Zhengyou Zhang

**Affiliations:** ^1^Microsoft Research, Redmond, WA, United States; ^2^Division of Biology and Biological Engineering, California Institute of Technology, Pasadena, CA, United States; ^3^UCL Interaction Centre, University College London, London, United Kingdom; ^4^Interactive Systems DEI-Lab, Universidad Carlos III de Madrid, Madrid, Spain; ^5^Department Electrical Engineering, University of Washington, Seattle, WA, United States

**Keywords:** virtual reality, HRTF (head related transfer function), spatial audio, auditory perception, auditory training, cross-modal perception, cross-modal plasticity

## Abstract

Auditory spatial localization in humans is performed using a combination of interaural time differences, interaural level differences, as well as spectral cues provided by the geometry of the ear. To render spatialized sounds within a virtual reality (VR) headset, either individualized or generic Head Related Transfer Functions (HRTFs) are usually employed. The former require arduous calibrations, but enable accurate auditory source localization, which may lead to a heightened sense of presence within VR. The latter obviate the need for individualized calibrations, but result in less accurate auditory source localization. Previous research on auditory source localization in the real world suggests that our representation of acoustic space is highly plastic. In light of these findings, we investigated whether auditory source localization could be improved for users of generic HRTFs via cross-modal learning. The results show that pairing a dynamic auditory stimulus, with a spatio-temporally aligned visual counterpart, enabled users of generic HRTFs to improve subsequent auditory source localization. Exposure to the auditory stimulus alone or to asynchronous audiovisual stimuli did not improve auditory source localization. These findings have important implications for human perception as well as the development of VR systems as they indicate that generic HRTFs may be enough to enable good auditory source localization in VR.

## Introduction

How we identify the source of sounds in space is determined largely by three acoustic cues: (a) interaural time differences (ITD), (b) interaural level differences (ILD), as well as (c) acoustic filtering i.e., spectral cues derived from the shape of one's ears, head, and torso (Møller et al., 1995; Majdak et al., 2014). Together, these cues provide us with a fairly accurate representation of acoustic space (Sabin et al., 2005). To simulate natural acoustic perception in Virtual Reality (VR) these auditory spatial cues are usually rendered using Head Related Transfer Functions (HRTFs), which can either be generic or individualized. The use of HRTFs leads to accurate source localization and increased sense of presence within the virtual environment, when compared to non-spatialized audio (Hendrix and Barfield, 1996; Bergstrom et al., 2017). Individualized HRTFs are calibrated on a per user basis, and are therefore better suited to simulate one's natural acoustic environment. However, creating individualized HRTFs can be very time consuming, technically difficult, and expensive to implement (Meshram et al., 2014). On the other hand, generic HRTFs can be pre-calculated which makes it easier to deliver spatialized sound to any device with head tracking (Gardner and Martin, 1995). Unimodal comparisons between auditory source localization of virtually rendered sounds using generic vs. individualized HRTFs have revealed that the use of generic HRTFs leads to increased confusion over auditory source location (Wenzel et al., 1993) and an increase in the magnitude of source localization errors (Middlebrooks, 1999). Thus, improving the perceptual experience of generic HRTFs could be enormously beneficial to remove the current practical barriers associated with individualized HRTFs.

Research on auditory perception suggests that our representation of acoustic space is fairly plastic (Fiorentini and Berardi, 1980; Shinn-cunningham et al., 1998; Seitz and Watanabe, 2005; Keuroghlian and Knudsen, 2007; Carlile, 2014). Manipulating acoustic cues by blocking one ear has shown modest improvements in auditory spatial localization over a period of 2–7 days (Bauer et al., 1966; Kumpik et al., 2010). Subsequent research has investigated auditory performance in response to altered ITDs using generic HRTFs. In these experiments the researchers found that the participants' auditory localization performance improved following a series of training sessions repeated of 2–6 weeks (Shinn-cunningham et al., 1998). While these findings demonstrate improved localization performance following unimodal training, the long exposure periods required for only limited improvements, make this an impractical solution to improving the perceptual experience for casual users of generic HRTFs.

Research on multisensory integration (Witten and Knudsen, 2005; Ghazanfar and Schroeder, 2006; Stein and Stanford, 2008) and multisensory learning (Shams and Seitz, 2008; Paraskevopoulos et al., 2012; Connolly, 2014) have highlighted the extent to which visual perception can influence auditory perception (Howard and Templeton, 1966; Vroomen et al., 2001; Bonath et al., 2007) and even lead to rapid changes in one's acoustic perception (Recanzone, 1998; Lewald, 2002; Wozny and Shams, 2011). One classic example of the visual influence over the perceived location of sounds can be observed in the ventriloquist illusion—an audiovisual illusion in which the perceived location of an auditory source is translocated toward a visual source that is presented at the same time, but in a different location (Howard and Templeton, 1966; Bertelson and Aschersleben, 1998). Moreover, it has been found that repeated exposure to the ventriloquist effect can lead to a “ventriloquism after-effect” in which spatially disparate but temporally aligned audiovisual stimuli lead to an altered representation of acoustic space (Recanzone, 1998; Woods and Recanzone, 2004; Frissen et al., 2005, 2012). That is, a visual stimulus presented slightly to the right of the veridical source of the auditory stimulus leads to a remapping of acoustic space. This will cause individuals to misperceive auditory stimuli as coming slightly to the side of their veridical sources when presented alone (i.e., without visual stimuli). Similar visual-to-auditory adaptation effects have been observed for the representation of auditory motion. Kitagawa and Ichihara (2002) found that repeatedly viewing visual objects moving in depth led to an auditory aftereffect in which spatially static sounds were miss-perceived as moving in the opposite direction (Kitagawa and Ichihara, 2002). Together, the findings presented above highlight the highly adaptable nature of the auditory system, and the importance of vision in shaping acoustic perception (cf., Berger and Ehrsson, 2016). Given the known plasticity of the auditory system, and the importance of vision in generating rapid changes in acoustic perception, research and development of HRTFs in VR could be significantly improved by applying some of these basic principles of human sensory perception.

Here, we examine whether it is possible to recalibrate users' auditory perception to a new virtual acoustic environment, rather than adapting the environment to the users inside VR. We sought to investigate whether brain plasticity mechanisms can be exploited via cross-modal learning from vision to improve auditory source localization. Using generic HRTFs, we first examined whether exposure to spatially and temporally aligned audiovisual (**AV**) stimuli would improve subsequent auditory-only source localization. In a control condition (**Auditory Only**), we examined whether exposure to the auditory stimulus alone would also improve auditory-only source localization. In an additional follow-up experiment, we further explored whether the introduction of an impact auditory stimulus associated with the physics of the moving visual object would strengthen any observed AV-driven improvement in subsequent auditory source localization (**AV + Impact Sync**) and whether temporally dissociating the audiovisual stimuli would prevent any subsequent improvement in source localization (**AV + Impact Async**). Consistent with previous research on the plasticity of the auditory system we hypothesized that exposure to spatio-temporally congruent AV stimuli within the virtual environment would lead to a spatial recalibration of acoustic space and therefore improve the participants' subsequent auditory-only source localization. On the contrary, exposure to the auditory stimuli alone or asynchronous AV stimuli would not. To further examine the generalizability of the remapping of acoustic space from one sound-type to another, we performed an additional experiment in which different sounds were used for the localization test stimuli and the training stimuli (**V + Impact Sync**).

## Materials and methods

### Experimental design and stimuli

**Figure 1:**
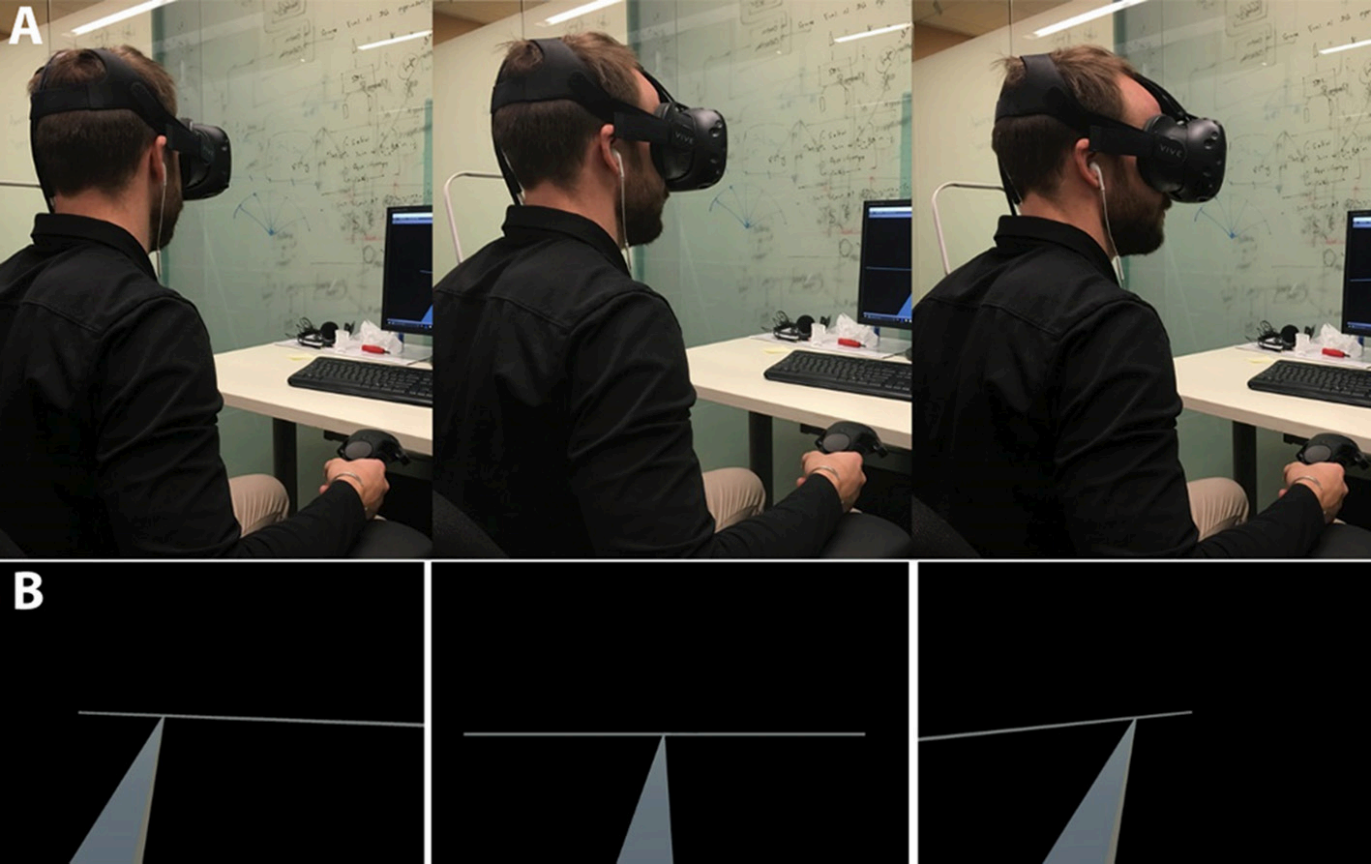
Experimental setup. **(A)** The participants were equipped with the VR headset and could identify and report the source of sounds originating from five different locations (± 26.6°, ± 11.3°, 0°) along a white bar that was located 10 m in front of the participant and spanned 73.74° along the azimuth. **(B)** First person perspective within the VR environment during the auditory localization task. (The person in the picture is an author of the paper and gave consent to publish an identifiable image of him).

The current paper includes a series of experiments that were presented to the participants in three phases:

1. Pre-exposure auditory source localization test. During the pre-exposure phase participants performed a localization test. This test consisted of identifying the source of a repeating “beep-like” sound presented at 55 dB SPL via in-ear headphones (frequency range = 0.042–15.21 kHz, duration = 190.5 ms; rise/fall time = 15 ms; silent interval between repeat = 19.6 ms). Participants used a white cylinder attached to their head, i.e., virtually linked to the head mounted displays (HMDs), and projected outwards in space to point to the perceived source of the sound and used the hand-held trigger to log their response and proceed with the next trial (see Figure 1). The repeating beep tone came from one of 5 different locations (±26.6°, ±11.3°, 0°) along a horizontal white bar situated 10 meters in front of the participants along the azimuth (visual angle = 73.74°). The pre-exposure phase of the experiment consisted of 25 trials (5 trials per auditory location). Trials were presented randomly.
2. Exposure phase. The exposure phase had a duration of 60 s and consisted of an auditory source moving in 3D space (see Video V1). The audio sound during this phase was the same “beep-like” auditory stimulus used in the localizationphases (frequency range = 0.042–15.21 kHz, duration = 190.5 ms; rise/fall time = 15 ms; silent interval between repeat = 19.6 ms). The audio source was sometimes co-located with a visual stimulus (AV and AV + Impact), orpresented unimodally (without a visual counterpart; i.e., Auditory Only), depending on the experimentand the condition, as explained below:
  - In the main experiment (**Unimodal vs. Multimodal mapping** experiment), we presented an audiovisual (**AV)** condition in which we attached a visual stimulus (white sphere, radius = 0.5 m; mean visual angle = 5.72°) to the auditory source. The hypothesis was that cross-modal learning would help to remap the acoustic space and improve subsequent auditory source localization in VR. In the **Auditory Only** control condition, there was no visual counterpart to the auditory motion. We designed this unimodal condition to rule out the possibility that any observed improvement in the localization of the AV condition could simply be due to improved accuracy over time, but due to the cross-modal influences.
  - In the second experiment (**Multimodal Mapping with Impact Sound** experiment) we introduced the **impact sound** conditions with additional bottom-up sounds associated with the physics of the moving object (i.e., an impact sound when the object abruptly changed direction) in the virtual environment (**AV + Impact Sync** condition). Previous experiments have shown that impact sounds can have very strong effects on audiovisual motion perception as they are naturally associated in a bottom-up fashion with related visual motion cues (Sekuler et al., 1997; Shimojo and Shams, 2001). Thus, in addition to the AV experiment's repeating beep-like sound, we also introduced an impact sound (with exponential decay starting at height = −5 dB and until −20 dB, duration = 150 ms frequency range = 0.042–18 kHz). This impact sound was spatially and temporally aligned with each visual bounce (i.e., abrupt change in direction) made by the white sphere. As a control condition in this experiment we manipulated the temporal relationship between the impact sound and the changes in direction of the white sphere, so that there was a random temporal delay of at least 300 ms between both stimuli (**AV + Impact Async**). Therefore, here we sought to examine whether introducing temporal asynchrony between the impact sound and the visual bounce during the exposure phase would reduce or abolish any improvements in auditory spatial acuity observed in the experiments above. Note that the temporal delay value was random but always chosen to fall outside of the temporal window for which auditory and visual stimuli may be perceived as simultaneous and multisensory integration can occur (Lewkowicz, 1996, 1999). The spatial relationship between the sphere and the sounds remained intact in this condition. That is, the sounds were still co-located spatially with the white sphere as it moved around in the 3D environment, but the impact sound played asynchronously with respect to the sphere's abrupt changes in trajectory. Adding this experiment with impact sounds allowed us to also explore the effects of temporal asynchrony on the cross-modal influences for auditory remapping.
  - In a third experiment (**Remapping with Impact Sound Only**) we used the impact sound as the sole audio stimulation during the AV Exposure Phase, while the localization test is still done using the beep-like stimulus (i.e., the test and the training are done in different sounds) (V + Impact Sync). The aim of this experiment is two-fold: on one hand, the results will provide evidence for whether the synchronous presentation of the impact sound with each bounce of the visual object during the adaptation phase is sufficient to recalibrate acoustic perception, and on the other hand, it will help provide evidence for whether the remapping of acoustic space in VR can easily transfer from one kind of sound to another. Previous work suggests that the remapping of acoustic space (outside VR) does not transfer across disparate frequencies (Recanzone, 1998). Therefore, the results from this study will have theoretical as well as practical significance for future work focused on improving auditory source localization of users of generic HRTFs.
3. Post-exposure auditory source localization test: Following the exposure phase, participants once again performed the sound localization test, consisting of 25 trials. The stimuli and procedures for the post-exposure phase trials were identical to the pre-exposure phase across all the experiments.

At the beginning of the experiments participants answered a demographic questionnaire.

#### Participants

Seventeen participants were recruited to participate in the unimodal vs. multimodal mapping experiment (mean age = 37.1 years, *SD* = 9.4; 4 females). Sixteen participants were recruited for the Multimodal Mapping with Impact Sound experiment (mean age = 37.3 years, *SD* = 9.7; 4 females). Eleven participants participated in the Remapping with Impact Sound Only experiment (mean age = 34.2 years, *SD* = 6.8; 5 females). The conditions on each experiment were counterbalanced and presented to all participants in a within subject design. There was at least 1 day of rest between conditions. All participants were recruited from within Microsoft Research, were healthy, reported no history of psychiatric illness or neurologic disorder, and reported no impairments of hearing or vision (or had corrected-to-normal vision). The experimental protocol was approved by Microsoft Research and followed the ethical guidelines of the Declaration of Helsinki. Participants gave written informed consent and received a lunch card as compensation for their participation.

### Apparatus

All visual stimuli were presented via an HTC Vive HMD with a 110° FoV and 2160 × 1200 combined resolution for both eyes (refresh rate = 90 Hz) and equipped with a position tracking system. Both the head tracking and the controller positions and rotations were acquired using the HTC Vive system based on lighthouses that implement laser LIDAR technology with sub-millimeter precision. The head tracking enabled the spatialization of the audio in real-time based on the user's current head pose using a generic Head Related Transfer Function (HRTF), based on the KEMAR data set (Gardner and Martin, 1995), which preserved the sensorimotor contingencies for the audio motor perception. Sounds were presented through in ear-headphones (model Earpod). During the auditory localization tests participants used a HTC Vive hand-held remote to log their response and proceed with the next trial once they were confident that they were pointing with the HMD at the correct location of the sound. Stimulus presentation and data collection were controlled using Unity 3D Software (version 5.3.6f1).

### Statistical analyses

We ran a statistical analysis to examine whether a 60-s exposure to the dynamic stimulus moving around in 3D space could improve auditory source localization. For each sound localization trial, we calculated the intersection of the ray projected from the participants' head and the horizontal line from which the sounds originated along the azimuth in 3D space. The spatialization error was then calculated as the distance between this location and the true source of the sound. The process was completed for each location for each trial, and then averaged across locations and trials for each participant for the pre-exposure and post-exposure phases, separately.

We then ran paired comparisons between the pre-exposure and post-exposure localization error scores in order to measure the pre-post improvement for each experiment. In all cases, a Shapiro-Wilk test was run prior to conducting the pair comparisons to confirm the assumption of normality in the paired-differences between the pre-exposure and post-exposure errors. For the cases when the normality assumption was fulfilled, we ran a paired *t*-test.

For the cases in which within-subjects' analysis was available (AV vs. Auditory only, and AV + Impact Sync vs. AV +Impact Async) we ran a repeated measures ANOVA. Test of Statistical Equivalence (TOST) was performed to find similar distributions among the data. All statistical analyses were performed using the computing environment (R Core Team, 2016). The data for this study have also been made available online (see Data Sheet 1 in Supplemental Materials).

## Results

### Unimodal vs. multimodal mapping

**Figure 2:**
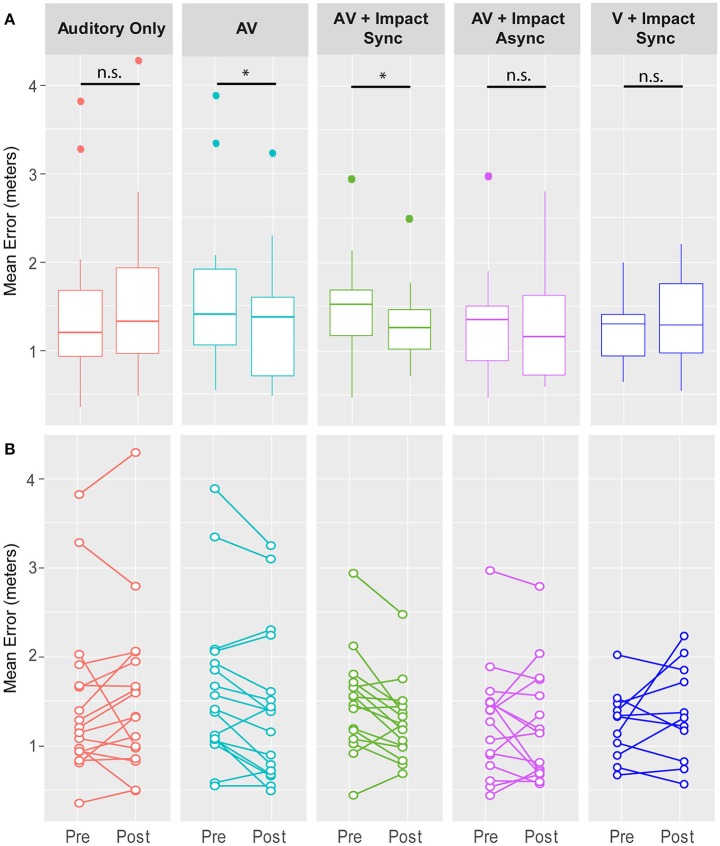
Results from all experiments. **(A)** Box-plots of the auditory remapping for all experiments. A significant improvement of the participants' auditory localization error wasand the localization test in a observed following the 60 s Audiovisual (**AV**) exposure. No such improvement was observed following the **Auditory Only** exposure. In the experiment on the effect of impact sounds, improved auditory source localization was observed following the synchronous audiovisual exposure phase with the additional impact related auditory cues (**AV + Impact Sync**). No significant remapping was observed following exposure to asynchronous but spatially aligned audiovisual stimuli (**AV + Impact Async**), or when the training was done in one sound and the localization was tested using a different sound **(V + Impact Sync)**. **(B)** Mean pre- and post- adaptation localization errors for all participants, with each participant's data represented by pair of dots connected by a line. Asterisks indicate significant difference between pre-exposure and post-exposure phases (^*^p < 0.05) and “n.s.” indicates that there was no significant difference between pre- and post-exposure phases (*p* > 0.05).

The results from the first experiment compared Audio only to AV remapping (Figure 2). Repeated Measures ANOVA with factors Condition (Audio, AV) × Test (pre, post), showed a significant within subjects interaction between Condition and Test [*F*_(1, 16)_ = 5.62, *p* = 0.03, $\eta_{p}^{2}=\;0.26$]. Planned comparisons of pre- and post-exposure conditions, revealed that the synchronous moving audiovisual stimulus in the 3D environment significantly reduced the participants auditory source localization errors, *t*_(16)_ = 2.87, *p* = 0.011, $\eta_{p}^{2}=\;0.34$, 95% CI [0.05, 0.35]. That is, localization accuracy was significantly better during the post-exposure phase (localization error: *M* = 1.41, *SD* = 0.85) compared to the pre-exposure phase (localization error: *M* = 1.61, *SD* = 0.88). However, the remapping effect was not found after exposure to only the moving sound, as the comparison between the pre- and post-exposure Audio only conditions was not significant {*t*_(16)_ = 0.4, *p* = 0.53, $\eta_{p}^{2}=\;0.02$, 95% CI [−0.28, 0.15]}. These results indicate a stronger auditory accuracy improvement in the AV condition than in the Audio only condition. A Shapiro-Wilk test for normality confirmed that the paired-differences between the pre-exposure and post-exposure errors in both the AV and Audio only conditions did not violate the assumption of normality (*W* = 0.95, *p* = 0.634 and *W* = 0.95, *p* = 0.417, respectively).

### Multimodal mapping with impact sound

We also examined whether the addition of an impact sound associated with each change in the visual stimulus' direction in the environment would further improve the auditory spatial remapping (AV + Impact Sync condition). This manipulation also allowed us to examine whether disrupting the temporal relationship between the visual object and an associated sound would reduce the cross-modal recalibration effect (AV + Impact Async Condition). The AV + Impact Async condition kept the spatial relationship between the sphere and the auditory stimulus the same, and manipulated only the temporal correspondence between the bounce and the occurrence of the impact sound.

We ran a within subjects repeated measures analysis with factors Test (pre, post) × Condition (AV + Impact Sync, AV + Impact Async) and found a significant within subjects interaction in Test × Condition *F*_(1, 15)_ = 7.625, *p* = 0.01, $\eta_{p}^{2}=\;0.34$, (see Figure 2). Planned comparisons of localization performance between the pre- and post-exposure phases of the AV + Impact Sync condition revealed a significant reduction in the participants' auditory source localization error during the post-exposure phase (*M* = 1.29, *SD* = 0.42) compared to the pre-exposure phase (*M* = 1.50, *SD* = 0.55), *t*_(15)_ = 2.9, *p* = 0.011, $\eta_{p}^{2}=\;0.36$, 95% CI [0.05, 0.36]. However, the comparison between localization performance in the pre- and post-exposure phases in the AV + Impact Async condition was not significant {*t*_(15)_ = 0.24, *p* = 0.632, $\eta_{p}^{2}=\;0.01$, 95% CI [−0.15, 0.24]}. A Shapiro-Wilk test confirmed normality of the paired differences localization performance between pre- and post-exposure phases for both the AV + Impact Sync (*W* = 0.98, *p* = 0.97) and AV + Impact Async conditions (*W* = 0.99, *p* = 0.99).

An independent samples between subjects *t*-test revealed that there were no significant differences between the localization performance in the AV + Impact Sync condition and the AV condition from the previous experiment {*t*_(31)_ = 0.17, *p* = 0.9, $\eta_{p}^{2}=\;0.002$, 95% CI [−0.21, 0.20]}. Further, a Test of Statistical Equivalence (TOST) revealed that the localization performance in the AV Impact Sync condition and the AV Condition were equivalent (*df* = 18.9, *p* = 0.01, confidence = 0.97). However, the AV Impact Async Condition was not equivalent to the AV Impact Sync (rejected: *df* = 18.7, *p* = 0.14, confidence = 0.71).

### Remapping with impact sound only

We ran an additional experiment that examined the use of the impact sound as the only auditory cue during the AV Exposure phase (without the beep-like sound). As in all previous experiments, the post-localization test was done with the beep-like sound.

Planned comparisons of localization performance between the pre- and post-exposure phases of this experiment revealed no significant reduction in the participants' auditory source localization error during the post-exposure phase (*M* = 1.22, *SD* = 0.11) compared to the pre-exposure phase (*M* = 1.35, *SD* = 0.16), {*t*_(10)_ = 1.012, *p* = 0.33, $\eta_{p}^{2}=\;0.01$, 95% CI [−0.16, 0.42]}. A Shapiro-Wilk test for normality confirmed that the paired-differences between the pre-exposure and post-exposure errors did not violate the assumption of normality (*W* = 0.89, *p* = 0.16).

## Discussion

In the experiments presented here, we have demonstrated that pairing a visual stimulus with an auditory source in virtual 3D space for a duration as short as 60 s is sufficient to induce a measurable improvement in auditory spatial localization in VR. The improvement did not occur when the moving auditory stimulus was not paired with a visual stimulus, or when the paired visual stimulus was temporally inconsistent (i.e., asynchronous audiovisual stimuli). Additionally, we found that the remapping does not transfer well if the training and the test were done with two different types of sound. Given these results, we believe that synchronous multisensory stimulation is key for a rapid adaptation to novel spatialized audio cues. Our results are consistent with previous findings suggesting that the brain can accommodate changes in acoustic mapping though multisensory learning (King, 2009; Carlile, 2014). Considering the improved auditory source localization within the VR environment, these findings support the use of multisensory recalibration techniques when utilizing generic HRTFs. Furthermore, we suggest that personalized HRTFs may not be required for users to experience accurate auditory source localization if they are able to recalibrate their auditory perception through cross-modal techniques.

The results from this study suggest that the improvement in auditory source localization through exposure with a paired visual stimulus occurs through multisensory integration processes. Remapping was stronger in the AV + Impact sound experiment when the auditory stimulus was presented in synchrony with an additional bounce-like sound consistent with the physics of the moving object, as compared to when the bounce-like sound was asynchronous, and thus multisensory integration was disrupted (Lewkowicz, 1996, 1999). This effect was found even though the auditory and visual stimuli were still spatially congruent in the environment, which suggest that either (a) noise in the environment can lead to a deterioration of visually induced improvements in auditory spatial acuity; and/or (b) top-down knowledge of the relationship between the visual and auditory stimuli are necessary to have a discernible effect on auditory source localization. Previous work suggests that it is likely a combination of both factors (Shimojo and Shams, 2001). The temporal relationship between sounds is critical for the low-level perceptual organization of sound early on in the auditory processing stream (Bregman, 1990), and also plays a critical role in identifying whether sounds are of the same or a different source (Pressnitzer et al., 2008). Additionally, research on top-down auditory source localization suggests that explicit attention and knowledge about the target auditory stimulus is also needed to segregate or group auditory stimuli in a noisy environment. These factors form the basis of the well-known “Cocktail party effect.” The “cocktail party effect” refers to the ability of hearing a specific sound of interest in a noisy environment (Cherry and Taylor, 1954; McDermott, 2009). Consistent with both research on bottom-up —i.e., low-level mediators of auditory scene analysis—and on top-down influences on perceptual grouping, the results from our study indicated that top-down knowledge of visual objects could be disturbed by bottom-up factors such as the temporal relationship between the visual and auditory stimuli (Sanabria et al., 2006). We found that asynchrony between the bounce-like sound and the visual stimulus (AV + Impact Async) did not significantly improve auditory source localization.

Additionally, when the impact sound was presented as the sole audio cue in the AV Exposure Phase, the training did not lead to an improvement in the post-localization test that was performed with a different type of sound (beep-like sound). This finding is consistent with previous work which has shown that the recalibration of acoustic space does not transfer between sounds of disparate frequencies/types (Recanzone, 1998; Frissen et al., 2005; Berger and Ehrsson, in press). Thus, our results suggest that the use of broadband noise or multiple sound-types should be used during the recalibration phase in future work aimed at utilizing AV recalibration as a means to improve auditory source localization for users of generic HRTFs. Our results also suggest that while the impact sound was able to disrupt AV binding and recalibration of the continuous beeping sound in the **AV + Impact Async** condition, it was not sufficient to recalibrate acoustic space for the beep sound on its own (in the **V + Impact Sync** condition) nor did it significantly improve recalibration in the **AV + Impact Sync** condition. This suggests that there is little to no perceptual benefit of additional acoustic cues (i.e., impact sounds) for the remapping of acoustic space for a given sound, and that a mismatch between such additional cues can only serve to disrupt the remapping of acoustic space.

Although in the current experiments we have addressed whether auditory spatial acuity can be improved from audiovisual training, we have only examined this effect along the horizontal plane. Further research should assess the effectiveness of AV recalibration on the front/back or up/down dimensions. This may be a particular area of interest for future research given that spectral cues provided by the geometry of the head, body, and ears are also crucial for spatially orienting sounds in these dimensions (Carlile et al., 2005; Carlile, 2014). Moreover, in this experiment, we have only used an exposure period of 60 s, as previous works have found that effects of audiovisual recalibration can be observed with this duration of exposure (Wozny and Shams, 2011; Frissen et al., 2012; Chen and Vroomen, 2013). However, additional research may serve to examine the minimal duration of AV training necessary for users to reach asymptotic localization performance. Furthermore, although previous work has demonstrated that auditory source localization is impaired when using generic HRTFs compared to individualized HRTFs (Mehra et al., 2016), and that even the use of individualized HRTFs can result in an increase in front-to-back confusion of auditory stimuli compared to free field localization (Wightman and Kistler, 1989), subsequent work has found that auditory source localization when using generic HRTFs can be as good as free field source localization performance (Wenzel et al., 1993) or individualized HRTFs (Romigh et al., 2017) after training. Thus, in light of our findings, future work will serve to directly compare auditory source localization performance when using individualized HRTFs vs. post-recalibration localization performance when using generic HRTFs. Additional work will also serve to explore the duration of auditory source localization improvements, and how much time is necessary to recalibrate to the real world after experiencing this new spatial acoustic mapping in VR.

Overall the experiments presented here provide new evidence in support of the high degree of cross-modal plasticity in cortical sensory processing. The psychophysical data indicate that the interaction between congruent auditory and visual stimuli is key to the spatial re-calibration of auditory stimuli in VR. Our research also opens new avenues for future visual and auditory motion studies. Inside VR, it is relatively easy to achieve and simulate dynamic systems that allow researchers to test spatialized multisensory integration (Väljamäe et al., 2008, 2009; Riecke et al., 2009; Padrao et al., 2016; Gonzalez-Franco and Lanier, 2017; Gonzalez-Franco et al., 2017). Thus, motivated by some of the recent advances on VR technologies, we put forth a new hypothesis that has the potential to improve the immersive experience when using generic HRTFs. We hypothesize that the improvement triggered by AV cross-modal plasticity in the audio spatialization might make generalized HRTFs potentially as good as individualized HRTFs. In which case, participants could undergo a non-invasive acoustic recalibration when they enter the VR, enabling them to rapidly adapt to the spatial cues provided by a multimodal combination of visual and auditory stimuli and thereby reducing the need for technologically complex and time-consuming pre-calibrations. Interestingly, our findings demonstrate that this re-calibration process does not require strenuous conscious effort or extensive training regimens on the part of the user. Placing congruent co-located visual and audio sources around the VR environment is sufficient to remap the auditory space and achieve higher spatialization accuracies.

## Author contributions

MG-F, CB, DF, ZZ: Designed the experiments; MG-F: Developed the rendering apparatus; CB: Prepared and ran the experiments; CB and MG-F: Analyzed the data; CB, MG-F, AT-J, DF, and ZZ: Discussed the data; CB and MG-F: Wrote the paper, AT: Provided critical revisions.

### Conflict of interest statement

The authors report their affiliation to Microsoft, an entity with a financial interest in the subject matter or materials discussed in this manuscript. The authors however have conducted the review following scientific research standards, and declare that the current manuscript presents a balanced and unbiased studies.

## Supplementary material

Video V1 The experimental procedure, setup, and conditions can be seen here: https://youtu.be/i97RvpXO0s4.
